# Railway underpass location affects migration distance in Tibetan antelope (*Pantholops hodgsonii*)

**DOI:** 10.1371/journal.pone.0211798

**Published:** 2019-02-04

**Authors:** Wenjing Xu, Qiongyu Huang, Jared Stabach, Hoshino Buho, Peter Leimgruber

**Affiliations:** 1 Conservation Ecology Center, Smithsonian Conservation Biology Institute, Front Royal, Virginia, United States of America; 2 Environmental Remote Sensing Laboratory, Department of Environmental and Symbiotic Science, Rakuno Gakuen University, Ebetsu, Hokkaido, Japan; Université de Sherbrooke, CANADA

## Abstract

Wildlife crossings are designed to mitigate barrier effects of transportation infrastructure on wildlife movement. Most efforts in evaluating crossing efficiency focus on counting animal use. However, crossings placed at suboptimal locations may alter animals’ natural movement pattern and decrease population fitness, which cannot be reflected solely by counts of animal use. The long-distance migration of Tibetan antelope (*Pantholops hodgsonii*) is directly affected by the Qinghai-Tibet Railway (QTR). Using the Wubei wildlife underpass along the QTR, we evaluated how underpass placement affects migration routes and decreases movement efficiency. We calculated the net-squared displacement of each animal to identify migration segments (wintering, calving, and migrating) based on Argos tracking data. We used two corridor modeling methods to identify optimal routes that theoretically require less energy to travel between seasonal habitats. We calculated the distance from actual migration routes recorded by Argos to the modelled optimal routes. We found that antelopes stray farther away from the optimal routes as they approach Wubei, indicating that animals have to deviate from their optimal migration pathway to access the railway underpass. On average, antelopes prolong their migration distance by 86.19 km (SEM = 17.29 km) in order to access the underpass. Our study suggests crossing location can affect animal migrations even if structures facilitate animal crossing. To better conserve long-distance migrations, long-term studies using tracking data which evaluate optimal migration routes are needed. We suggest considering the location and structural characteristics in designing and improving wildlife crossings, which do not only facilitate utilization, but also optimize animal movement processes such as migration.

## Introduction

Animal migration, the repetitive and predictable movement between spatially disparate habitat areas, is one of the most spectacular ecological phenomena on earth [1,2]. The timing, duration, and route that migratory species take are believed to be the result of long-term adaptations to spatially and temporally variable environmental conditions (e.g. weather and resource availability) [3,4]. The conservation of migration routes are critical to maintain population fitness of migratory animals [5]. When migration routes are disrupted, animals may deviate from optimal routes, increasing travel time and leading to higher energy expenditures, delayed arrivals, elevated predation risks, alterations in migratory behavior, and ultimately lead to population declines [2,6].

Linear transportation infrastructure, such as roads and railways, are often regarded as one of the principal threats to terrestrial wildlife migration worldwide, resulting in vehicle collision-related mortality, hampering access to traditional migration routes, and imposing barriers to optimal foraging [7–9]. To mitigate these negative effects, development projects are often required to create crossings to facilitate habitat connectivity. Decisions on where to place crossings, however, are often not informed by scientific evidence related to traditional migration routes [10]. When previous information on animal migration routes are unavailable, it is paramount to monitor and evaluate the efficacy of existing crossings in order to adaptively improve and make recommendations for the placement of future structures. This study aims to reveal potential impacts of a railroad crossing in western China that bisects a historic ungulate migration.

Tibetan antelope *(Pantholops hodgsonii*) provide a valuable case study to examine effects of crossings on ungulate migration. The Hoh-Xil population is one of four migratory populations characterized by their hundreds-of-kilometers migration between the Hoh-Xil (or Kekexili) and Sanjiangyuan Nature Reserves [11]. The Tibetan antelope migration is synchronized with their reproductive cycle and almost all long-distance migrants are females [12]. They depart from wintering sites to calving sites in May and return with their newborns [11,12]. Disturbances on migration are likely to affect population demographics disproportionally and have critical impacts on sustaining populations.

The Qinghai-Tibet Railway (QTR) formally began operations in 2006, stretching along the boundary between Hoh-Xil and Sanjiangyuan, resulting in a distinct separation of the seasonal ranges of the Hoh-Xil population. The total environment mitigation investment of the QTR project is claimed to be over 220 million dollars, which includes a total of 15 railway crossings built within the Hoh-Xil–Sanjiangyuan segment by the China Railway Corporation to preserve landscape connectivity [13]. Although no tracking record exists on the antelope migration before the construction of the Hoh-Xil section of QTR in 2001, post-construction monitoring document thousands of antelopes using the crossings, with rate of use increasing over time [13,14].

However, two issues are revealed from the post-construction monitoring. First, among antelopes using crossing structures, 100% of the westward animals and 97% of the eastward animals use one single crossing, the Wubei underpass (35°15'2.71"N, 93° 9'45.12"E) [14]. Second, observations along the QTR have found that antelopes wander along the fenced railway before crossing, suggesting disruption exists to the animals’ natural migration behaviors [15,16]. To date, the QTR crossings have only been evaluated by counts of use. Albeit a record of successful crossing events, counts of use do not reflect animal crossing efficiency [17,18]. Thus, further considerations of the effect of the underpass on migration patterns are needed to further examine the performance of QTR crossings in facilitating migratory connectivity.

We hypothesize that the Wubei underpass is not ideally located even though it is frequently used by animals, requiring animals to deviate from optimal migration routes and travel longer distance to find the possible crossing location. We model the optimal migration routes and test whether the placement of the Wubei underpass has impacted migration efficiency, measured by the distance between the optimal migration route and the actual migration route recorded by Argos satellite tracking devices. We define the optimal route as the route that requires the least energy expenditure according to the topographic landscape. We examine how antelopes’ distance to modelled corridors change as they approach the railway, and how the actual migration distance differ from the length of modeled optimal routes.

## Materials and methods

### Study area

**Fig 1:**
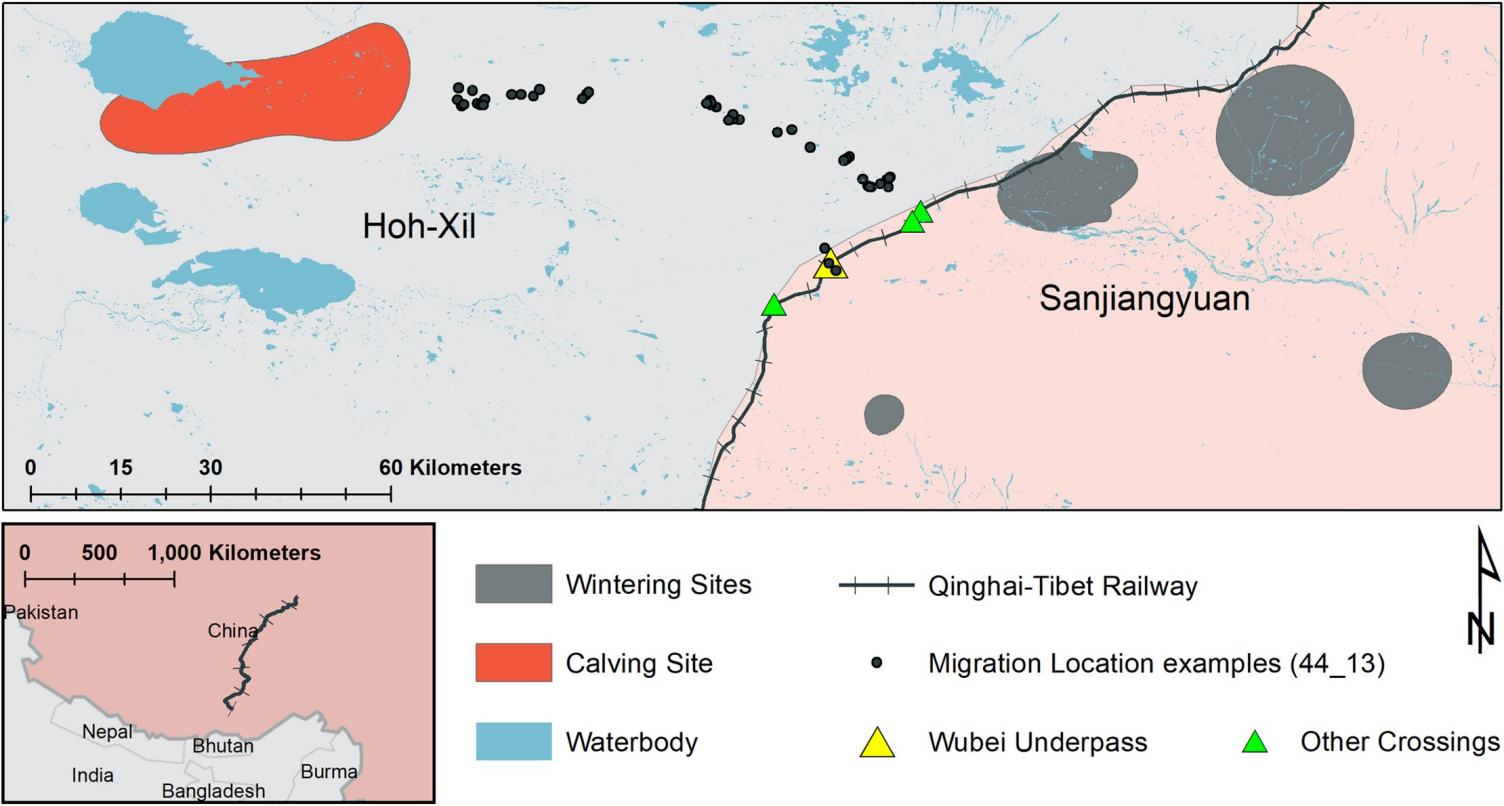
Study area in Qinghai, China with an example of Argos locations of one antelope-year demarcating the actual migration route. Winter and summer ranges are based on 50% fixed kernel density estimates of Argos locations. Four wintering sites are FR–Far range; MT–Mountain range; CR–Close range; and RV–River range.

Our study area (45,513 km^2^) is located in Qinghai, China. The elevation ranges from 4197 m to 5873 m. The QTR divides the study area along the boundary between the two National Nature Reserves: Hoh-Xil to the east (49,418 km^2^) and Sanjiangyuan (303,897 km^2^) to the west (Fig 1). The landscape is dominated by high-altitude steppe, alpine meadow, and gravel-filled gullies [6,19], and is one of the most remote regions in China. Human access is highly restricted. Tibetan antelope, mostly adult females, migrate from multiple wintering sites in Sanjiangyuan to a shared calving area in Hoh-Xil in late May or early June [12]. In July–August, animals return with their newborn calves [11]. Previous studies and observations show that Tibetan antelopes have high fidelity to the migration route [12].

The QTR bisects the migration route of Tibetan antelope approximately 40 km from their summer calving area. Four major crossing structures exist along our study area (Wubei underpass, Chumaer Bridge I and II, and Wudaoliang Bridge), all of which have been designed to facilitate migratory connectivity (Fig 1). Except for the crossing locations, the QTR Hoh-Xil section is fully fenced. Crossing structures offer the only means for antelope to cross the railway. Monitoring of the crossings show that antelope almost exclusively use the Wubei underpass, a 198-m long, 30-m wide structure [14]. We, therefore, focus on the Wubei underpass in this study.

### Argos tracking data analysis

Antelope location points were collected on 10 female Tibetan antelope of the Hoh-Xil population using Argos satellite transmitters between 2007 and 2014 (Model ST-20 A-3210, Telonics Inc., USA). The study was permitted by the State Forestry and Grassland Administration of China and the captures were conducted in collaboration with Shaanxi Institute of Zoology (Northwest Institute of Endangered Zoological Species). Raw data (6161 locations) were received and pre-processed by the Remote Sensing Laboratory at Rakuno Gakuen University, Japan. Data frequency was irregular, ranging from 0 to 3 points daily (for a detailed data description, see Buho et al., 2011 [15]. We retained locations with spatial errors < 1.5 km (location class 3, 2, and 1). For each individual, years with > 2 months of continuous data missing were removed in order to retrieve yearly migration cycles (additional details on data quality are provided in S3 Appendix). Our final dataset consisted of 896 points from 6 individuals and across 3 different years (2010, 2011, and 2013), constituting 8 antelope-year combinations (S1 File). All of the 8 migration cycles show locations on both sides of the railway in starting and returning trips, indicating successful crossings between two sides of the railway. Although the retained sample size is limited to 6 individuals, this dataset is the first and only telemetry dataset to date to document a complete migration route of the species of Tibetan antelope.

To identify the migration pattern of each individual for each year, we segmented location points using net-square displacement (NSD) [20,21]. In one migration cycle, the NSD of each location to the starting location can be calculated. The NSD value will reach a plateau when the individual moves to the other seasonal range. As the individual returns, the NSD decreases until reaching 0 as the individual returns to its starting location [21].

Each antelope-year was fit into a nonlinear mixed-effect model framework:

$$NSD=\frac{\delta }{1+\exp\left(\frac{\theta_{s}–t}{{\varphi_{s}}_{}}\right)}+\frac{-\delta }{1+\exp\left(\frac{\theta_{r}–t}{{\varphi_{r}}_{}}\right)}$$ (1)

where the model outputs are the estimations of squared migration distance (δ), timing of start/return (θ_s_/θ_r_), duration of the start/return trip (φ_s_/φ_r_), and the day of year (t). We classified locations before θ_s_ and after θ_r_ as wintering. Locations between θ_s_+φ_s_ and θ_r_-φ_f_ were classified as calving. Remaining locations were classified as migration. The NSD shows that all antelope shared a common summer calving site, whereas there are 4 wintering sites: Mountain Range (MT); Close Range (CR); River Range (RV); and Far Range (FR) (Fig 1). Migration points that fall within 95% fixed kernel density estimations [22] of summer and winter range are reclassified as calving and wintering, respectively. All migration points were located to the west side of the railway.

### Migration corridor modeling

We used two resistance-based methods to model the optimal, least energetically expensive migration corridors: least-cost path and circuit theory. The least-cost path approach identifies the path of least accumulated resistance between two points across a resistance surface [23]. This approach generates one single path connecting the start and end points, with the path being a single pixel (30-m) wide. As an alternative, circuit theory treats the landscape as an electrical-resistance surface and identifies multiple paths of current flow between habitat patches [24]. Least-cost paths are a more restrictive way to generate corridors, with the accuracy dependent on the accuracy of resistance surface. On the other hand, circuit theory considers all possible pathways and offers a flexible way to define corridor by setting different cutoff thresholds (the 100% corridor would cover the entire landscape, while the 0% corridor would cover no pixel at all).

As a first step, we created resistance surface and utilized the same surface for both corridor modeling methods. Resistance values on the surface approximated the physiological cost for antelopes to migrate between seasonal habitats. We calculated resistance values across the landscape based on waterbody locations, elevation, and degree slope. Waterbodies were extracted from the Globeland30 waterbodies dataset (http://www.globeland30.org). Elevation and degree slope were obtained from the ASTER global elevation dataset (http://earthexplorer.usgs.gov). Both datasets have a 30-meter resolution.

Elevation ranges were segmented into 5 groups (< 4000 m, 4000–4200 m, 4201–4500 m, 4501–5000 m, and > 5000 m) and assigned values of 25, 0, 50, 70, and 100, respectively. The weighting of different elevation ranges reflects the preferences of Tibetan antelope [25,26], with antelope preferring elevation ranges between 4000–4200 m. Slope (0° - 90°) was scaled to 0–100. The final resistance surface was calculated as the per pixel average of slope and elevation, with all waterbody locations assigned to the maximum resistance value (i.e., 100) since antelope movement is restricted by water. Although rivers in the Hoh-Xil area change seasonally, their volume is highest during the migration season, posting a consistent barrier to antelope movement. Thus, we did not consider river seasonality in our models. We also did not include measures of vegetation coverage or land-cover type, since Tibetan antelope do not strictly follow dynamics of vegetation nutritional state [19,27] and because no difference in vegetation type was observed across the study area (i.e., the entire area was steppe habitat).

The second step of corridor modeling is to identify core areas of antelope calving and wintering sites using 50% fixed kernel density estimates based on the NSD-classified calving and wintering Argos locations. These two areas were used as starting/ending areas to simulate migration corridors. We compared circuit theory corridors generated by 5%, 10%, 15%, 20%, and 25% current thresholds and chose 10% for analyses, since this threshold minimized the predicted area while providing a continuous habitat corridor (S1 Appendix). Both least-cost path and circuit theory corridors were generated in ArcGIS 10.3 [28].

To test corridor model sensitivity to resistance value calculations, we created two other least-cost path models based on two other resistance surfaces: one generated singularly from elevation, and one generated singularly from slope (S2 Appendix). A table summarizing all models generated in this study can be found in the supplementary materials (S1 Table).

### Analyzing underpass impacts

To test whether antelopes strayed from corridors when using the underpass, we first calculated distances from the Argos tracking data to the modelled corridors and to the underpass. In order to examine correlations, we fit linear models with distance from migration points to corridors as the response variable and distance to Wubei as the independent variable. Correlation was calculated using nonparametric Spearman’s ranking correlation coefficient. A negative correlation would indicate that antelopes stray farther from the optimal migration route as they approach the underpass. It is worth emphasizing that our intension was to show relative correlation, but not to parameterize the exact relationship between the two variables. Therefore, although spatial-temporal autocorrelation exists in the data points (it is inevitable for migration, a directional movement), our method is designed in a way that autocorrelation will not undermine our analysis or conclusion.

We also compared the observed and optimal migration distance to demonstrate whether using the underpass has prolonged the migration. First, observed migration distance was calculated in two ways: 1) extracting migration distance from the NSD estimations for each migration cycle; and 2) directly connecting Argos points. Since the temporal interval of Argos devices did not always capture locations near the railway, we treated the Wubei underpass as one recorded location and connected it with other Argos locations. This approach reflected the minimum distance that antelopes traveled when using the Wubei underpass. Second, the optimal migration distance was calculated as the length of the least-cost path for each wintering site. To confirm that the spatial error inherent in Argos telemetry data wouldn’t significantly bias the distance from optimal routes, we plotted the migration locations with a buffer indicating the spatial error relative to the least-cost paths and the Wubei underpass location (S3 Appendix). Analyses were conducted in R 3.3.2 [28].

## Results

**Fig 2:**
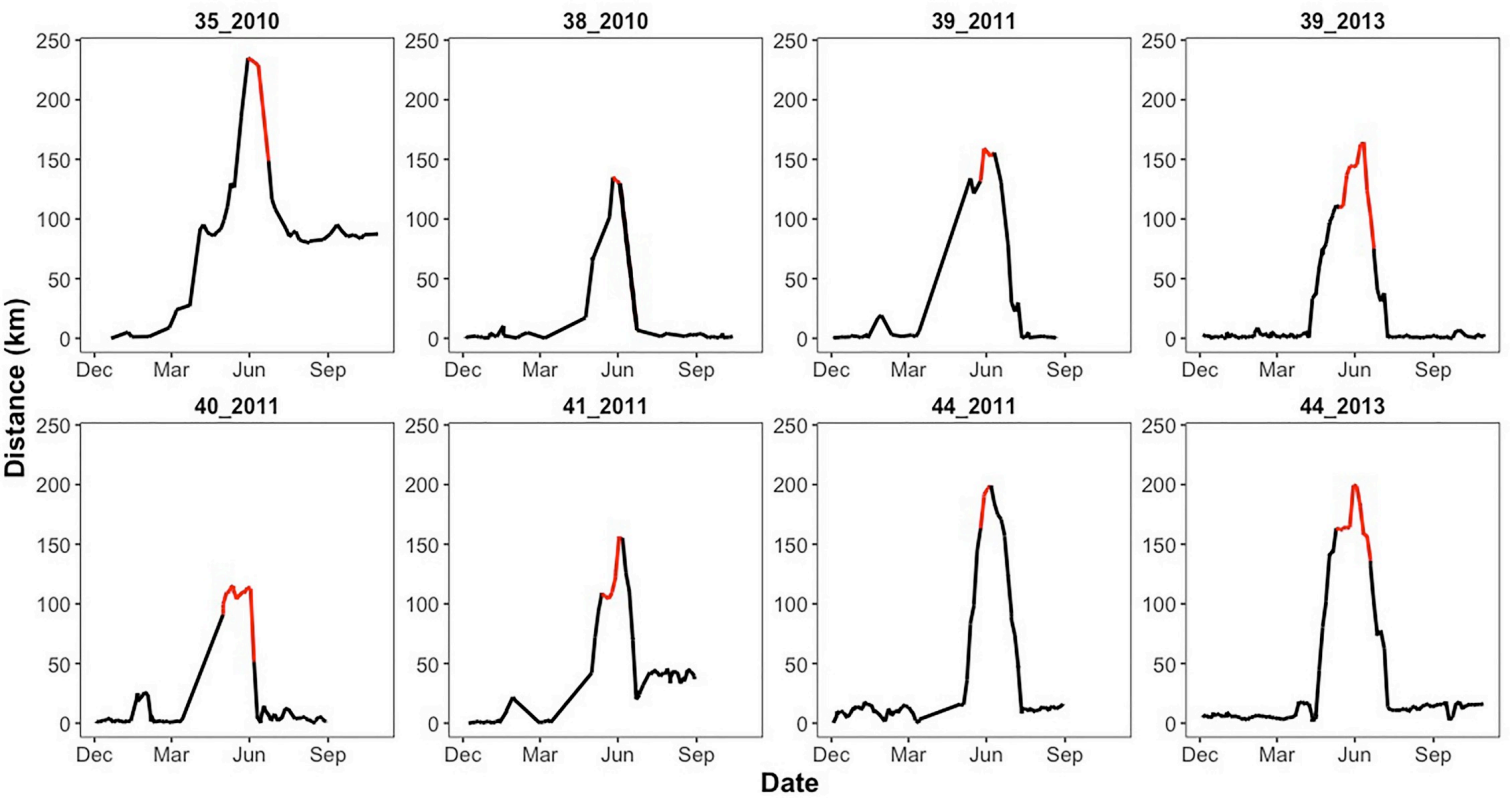
Net-square displacement (NSD) plot for each migration cycle of each individual Tibetan antelope (*Pantholops hodgsonii*) (individual ID_year). The NSD plot represents the distance to the starting point (wintering sites) where Antelopes stay at the beginning of a calendar year. During winter (the beginning of the graphs) the distance remain low until they depart for their calving site in late spring. They arrive at calving site in June when they reach the largest distance from the wintering site. Calving periods are represented by the peak of the graphs (highlighted in red), after which antelopes initiate the returning migration and distance starts to decrease. All antelopes except for 35_2010 and 41_2011 returned to their original wintering sites and the distances drop back to around 0.

Net-squared displacement revealed clear migratory patterns, with the highest NSD occurring during the calving period or on arrival/departure of calving sites (Fig 2). Four out of six individuals returned to their original wintering sites. Among the 8 antelope-year migration cycles, migration distance ranged on average from 163 km to 271 km, based on NSD estimations (S2 Table). Antelope began migrating on calendar day 162 (typically June 10th) and arrived back at their winter range on calendar day 198 (typically July 17th). Detailed NSD estimations are summarized in S2 Table.

**Fig 3:**
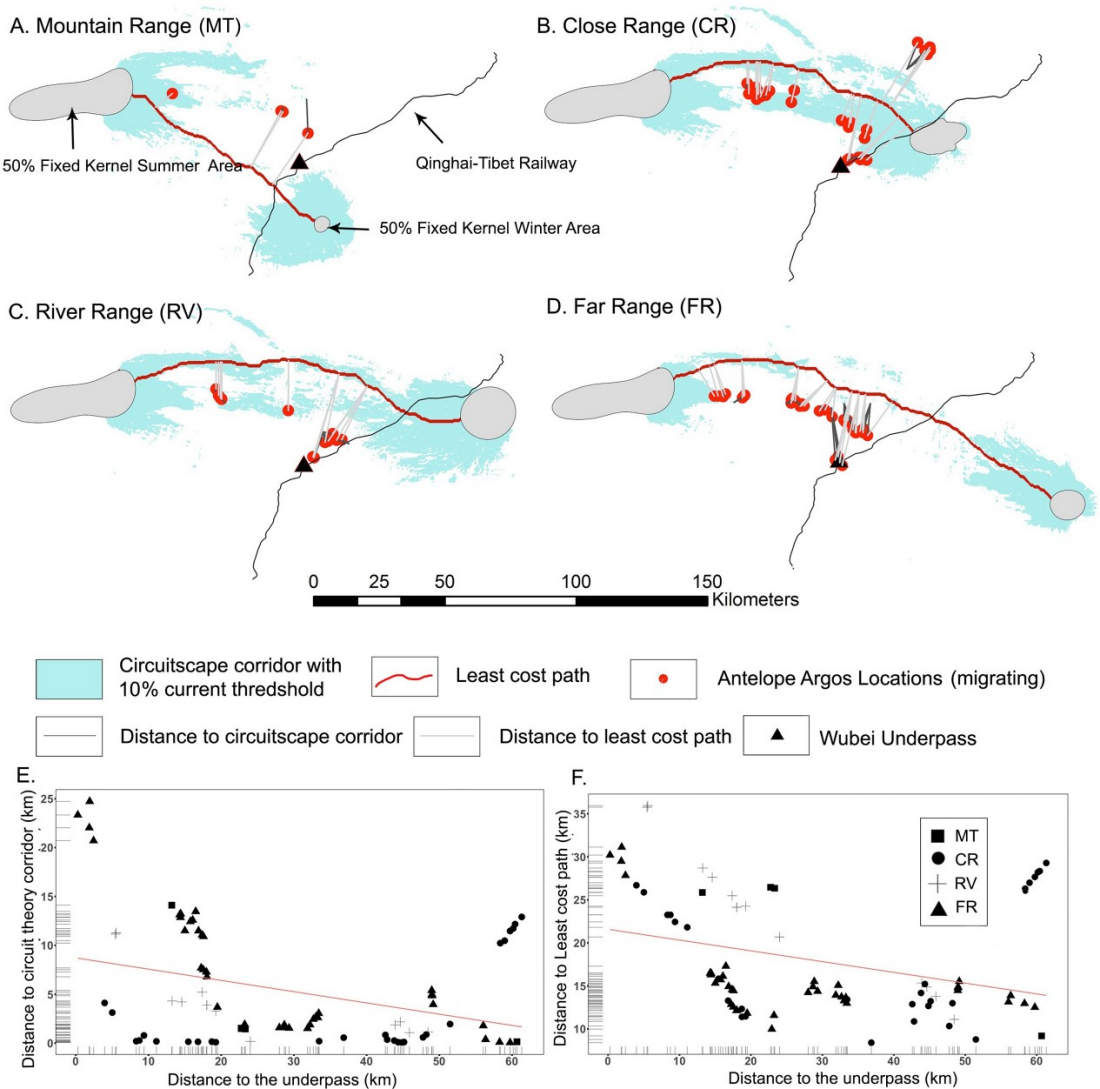
Modelled migration corridor and Argos locations of Tibetan antelopes. (A)-(D) Distance between Argos locations to modeled migration routes based on circuit theory and least-cost path, respectively, for each wintering site. (E) Distance to the underpass vs. Distance to circuit theory corridor. (F) Distance to the underpass vs. Distance to least-cost path.

A shared summer calving site and four separate wintering sites resulted in four starting/ending area pairs for antelope migration. We applied least-cost path and circuit theory modeling on each of the four data pairs (start and end points) and plotted them by wintering site (Fig 3A–3D). For the same wintering site, the circuit theory corridor and the least-cost path were generally in agreement, highlighting similar optimal pathways derived from each modeling methods. The Wubei underpass was not directly located along any of our modelled corridor migration scenarios (Fig 3A–3D).

Antelopes were found to stray from the optimal migratory pathway predicted by the model when approaching the railway underpass. This is demonstrated by the distance calculation that the closer antelopes are to the underpass, the farther they are away from the optimal routes (Fig 3E–3F). This trend is especially prominent in the circuit theory model (Fig 3E). When antelopes are more than 20 km away from the underpass, the circuit theory corridor represents the actual migration route well (i.e., most of the Argos locations are within 5 km from the modelled corridor). When within 20 km of the underpass, antelopes strayed away from the corridor. Since least-cost paths are a more restrictive way to estimate optimal corridors, antelope distance to least-cost paths are consistently larger than to the distance to the circuit theory corridor.

The correlation between the distance to the underpass and the distance to each of the corridor is consistent across the two modeling methods. Spearman rank coefficients highlight a negative correlation between antelopes’ distance from the underpass and their distance from the modelled corridors (Spearman r = -0.33, p < 0.01 for circuit theory corridor, Spearman r = -0.4, p < 0.01 for least-cost path).

**Fig 4:**
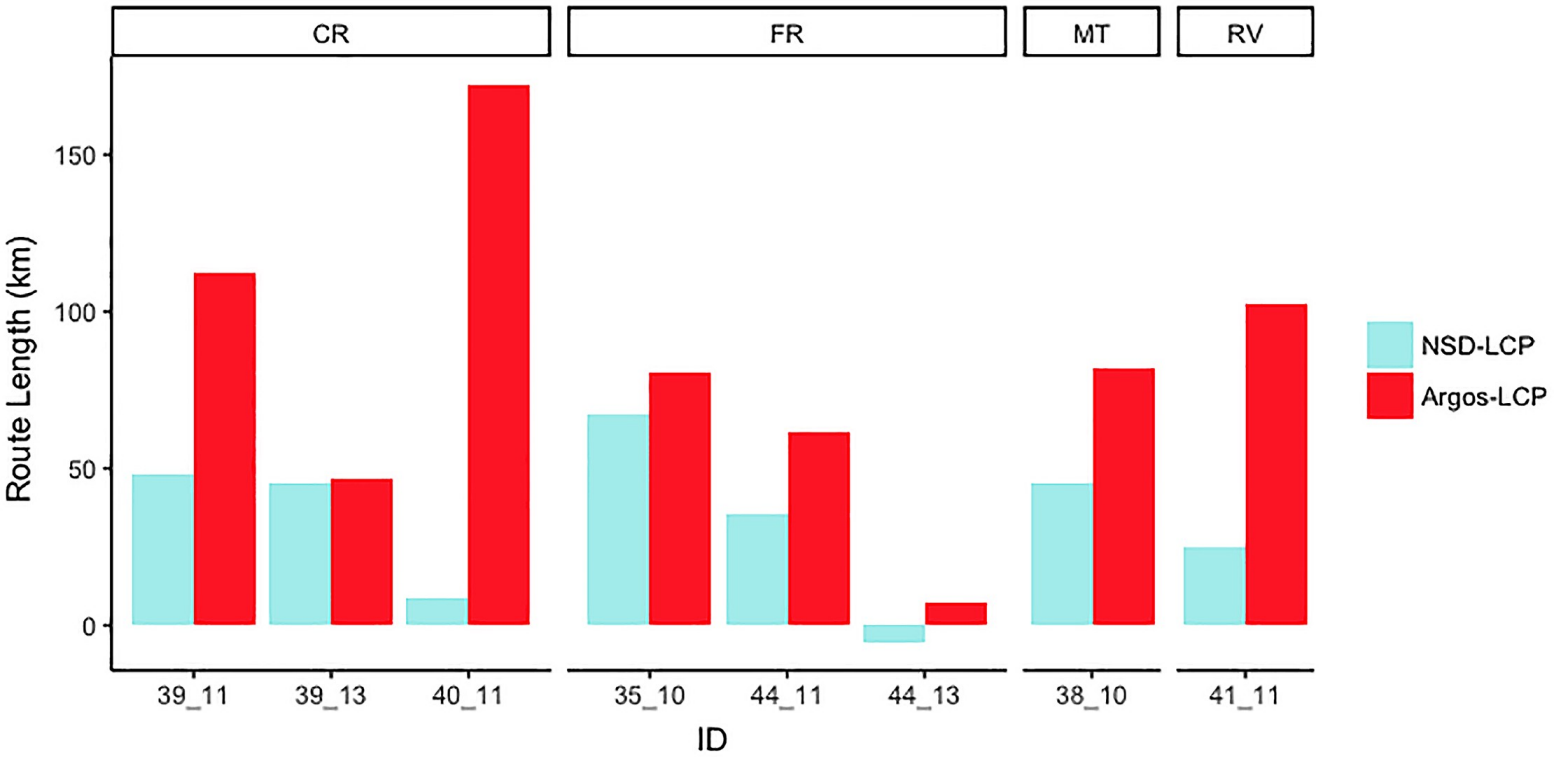
Extra distance that antelopes have to migrate in order to use the Wubei underpass. X-axis represents ID and is grouped by wintering site. Distances estimated by LCP represents optimal route lengths. Actual migration distances when crossing the railway via the Wubei underpass are estimated by net-squared displacement (NSD) and by directly connecting Argos locations with Wubei as one known point for each individual (Argos). The prolonged distances are calculated by subtracting LCP from NSD or Argos. The full table can be found in S3 Table.

Both methods of calculating observed migration distance show that antelope migrate longer distances than optimally determined migration routes. The NSD migration route is 33.75 km (SEM = 8.29 km) longer than the least-cost-path-estimated optimal migration route. When directly connecting Argos locations including the Wubei underpass, assuming antelope indeed used the underpass, the migration distance is lengthened by 86.19 km (SEM = 17.29 km) (Fig 4). This result confirmed our hypothesis that antelopes have to deviate from the optimal migration route and travel longer distance in order to use the crossing structure.

## Discussion

Wildlife crossings facilitate landscape connectivity by allowing animals to cross barriers without risk of traffic collision mortality. Such mitigation measures are widely installed in Australia, Europe, and North America [7,29,30]. QTR is the first railway project in China that implements wildlife mitigation measures in its design and construction. While the Wubei underpass does facilitate Tibetan antelope migration by allowing animals to cross the QTR, our results suggest that Wubei might not be located at the ideal location, leading animals to deviate from their optimal migration route in order to use the structure. This deviation was most prominent in the area closest to the underpass, which confirms our hypothesis that antelopes prolong migration route in order to cross the railway via the underpass. Such deviation from optimal migration routes led to increased distance traveled and greater energy expenditure.

In addition, disrupted migration patterns have the potential to put antelope population fitness and sustainability at risk. When animal migration is closely associated with reproduction, such as our case with Tibetan antelope, migration disruptions are especially detrimental during the return trip when lactating females must migrate to meet energy demands and feed their offspring [31]. Murray [32] found that lactating wildebeest (*Connochaetes taurinus*), for example, require 30% more energy per day than females in early pregnancy. Altered migration can also disrupt or unsynchronize breeding and reproduction cycles, leading to migrations that are uncoupled from vegetation phenology and lead to higher calf mortality [33]. Furthermore, prolonged migration distance can result in calving prior to arrival at traditional calving grounds. Manayeva et al. [19] has observed a 14 to 16 day delay in the starting time of Tibetan antelope migration since the railway was constructed, suggesting disruption of the temporal migration pattern.

Lastly, female and male antelope segregate their space use from January through October and almost all animals that migrate are females [12]. Disturbance on migration will thus disproportionally affect female antelopes and lamb. Such uneven impacts across demographics could be detrimental for population sustainability over generations. Therefore, even though the use of the crossing along the QTR has increased in recent years [13,34,35], overall persistence of the population may be negatively affected as a result of altered migration pathway and timing. Further studies on antelope fitness, recruitment, and demographic/behavioral changes are necessary to provide any long-term assessments of the effect of the increased migration distance on antelope populations.

Proper crossing design (e.g. location, width, length, and material) are critical to ensure the efficacy of mitigation structures [8,36]. Even though three other crossing structures exist in the study area, over 95% of antelope use the Wubei underpass [14]. Despite two other underpass structures (Chumaer Bridge I and Chumaer bridge II) being closer to the optimal migration routes, few antelope use them. This may be due to both bridges being constructed directly above waterways, making them impassable, especially for newborns during the migration season when water levels are high. The last underpass, the Wudaoliang bridge, is close to the Wudaoliang railway station (about 2 km) and is more likely to be affected by human activities. This leaves only the Wubei underpass. And while not ideally located, it is the best situated structure across the area. Accounting for the number of antelopes that use Wubei every year, this underpass provides the only viable connection between calving and wintering areas in this area.

Associated infrastructure, such as fencing, could also affect the efficacy of crossings. The QTR is fully fenced except for locations where crossings have been constructed. These fences prevent animals from crossing the QTR at locations other than the designated crossings. Previous studies observed that as antelopes approach the railway, they change directions and tend to move along the fence, searching for potential openings until they reach a crossing [15,16]. In searching for crossings, antelopes might stray further from optimal migration corridors. Over time, differential individual responses to fencing and crossing in the fragmented landscape could lead to population fragmentation. For example, Yu et al. [37] found genetic divergence of Przewalski’s gazelle (*Procapra przewalskii*) has been intensified since the construction of wired fences along the QTR, despite the existence of railroad crossings. Thus, assessment of impacts of associated infrastructure should also be considered in wildlife crossing evaluations.

Evaluating mitigation structure effectiveness is essential to ensure the usefulness of structures in maintaining population connectivity [38,39]. Most studies measure the effectiveness of crossings by summarizing the rate of use [40]. Counts of use, albeit a record of successful crossing events, do not reflect animal crossing efficiency [17,18]. In addition, such measures only focus on crossing events on the site of the structure, omitting the potential effects on the entire migration route. Thus, they are limited in ability to quantify impacts beyond the crossing location, such as prolonged migration, deviation from the optimal migration route, and lead to reduced migration efficiency [36,38]. We suggest that animal movement and behavioral studies be conducted before and after the construction of underpasses to reveal impacts of infrastructural development projects and the effectiveness of mitigation structures aimed to facilitate connectivity [10,41]. Most crossing studies, however, are limited by data availability and fraught with logistic difficulties [41].

With limited ability to conduct a before and after study, our study showcases a method to examine the indirect ecological impact of wildlife underpass through satellite tracking data and corridor modeling. This method could be widely applied to evaluate other structures that aim to mitigate obstruction to animal movement, especially for regions like the Tibetan plateau where data paucity is an issue [42]. Our study utilizes the first and only available Tibetan antelope tracking dataset to make comparison with two optimal corridor prediction models. Other complex methods, such as resource selection [43] or step selection functions [44], together with more accurate tracking technologies such as GPS collar, may offer a more empirical resistance surface for species that have specific habitat requirements during migration [45].

Once one of the most pristine regions in the world, the Tibetan plateau has been through severe social-economic and environmental changes in the past 50 years [46]. Development plans would benefit from having scientists involved at the early stages of design [10]. Comprehensive scientific research before and after construction are necessary to inform solutions that best balance the needs of both human and wildlife. Such studies are desirable, not just to enable crossing, but also to promote the efficiency required for animals to make the crossing. With limited environment monitoring data available for the Tibetan region and on Tibetan antelope [46], integrative, quantitative, and proactive scientific research is urgently needed to promote information justice of such remote region, and to preserve the important ecological phenomenon, long-distance migration, before they are lost.

## Supporting information

S1 Appendix Circuit theory corridors generated by 5%, 10%, 15%, 20%, and 25% current thresholds. (DOCX)

S2 Appendix Sensitivity analysis: least-cost paths based on different resistance surfaces. (DOCX)

S3 Appendix Spatial bias and data accuracy in Argos data. (DOCX)

S1 Table Summary of all corridor models generated in the study. (DOCX)

S2 Table Summary of migration parameters estimated from net-square displacement models. The model was fit in a nonlinear mixed-effect model framework for each individual in each year. Migration cycles are labeled using individuals’ ID and 2-digit year (“Individual_year”).
(DOCX)

S3 Table Migration distance (km) and detour distance for each individual-year migration cycle. Migration distance estimated by net-square displacement (NSD) and by directly connecting Argos relocations with Wubei underpass as one point represents actual migration conducted by antelopes. Distance estimated by least-cost path (LCP) represents optimal migration. Prolonged distance is calculated by subtracting LCP distance from NSD and Argos distance, respectively.
(DOCX)

S1 File Pre-processed Argos location. (CSV)
