# Supplementary material for: Railway underpass location affects migration distance in Tibetan antelope (Pantholops hodgsonii)
Source: PLoS One. 2019 Feb 4;14(2):e0211798. doi: 10.1371/journal.pone.0211798 (PMC6361455; doi:10.1371/journal.pone.0211798)
Supplement: S1 Appendix — (DOCX) [file pone.0211798.s001.docx]

# **S1 Appendix: Circuit theory generated by 5%, 10%, 15%, 20%, and 25% current threshold**

We created circuit theory maps for each of the four wintering sites (MT, CR, RV, and FR). Corridors were created by slicing pixels into the top 5%, 10%, 15%, 20%, and 25% values. Our goal for using circuit theory was to model the least cost path (i.e., the least energy demanding linear corridor) for antelopes to cross compared with other routes. Fig A.1 highlights that when the threshold is too small, only patches around destination sites are optimal. These models fail to form a connected corridor. When the threshold is too big, the model over-predicts the corridor area. Our aim was to select a small enough threshold to identify a contiguous corridor between the wintering and calving sites. Taking into consideration all four calving-wintering site pairs, a 10% threshold provided the best result.


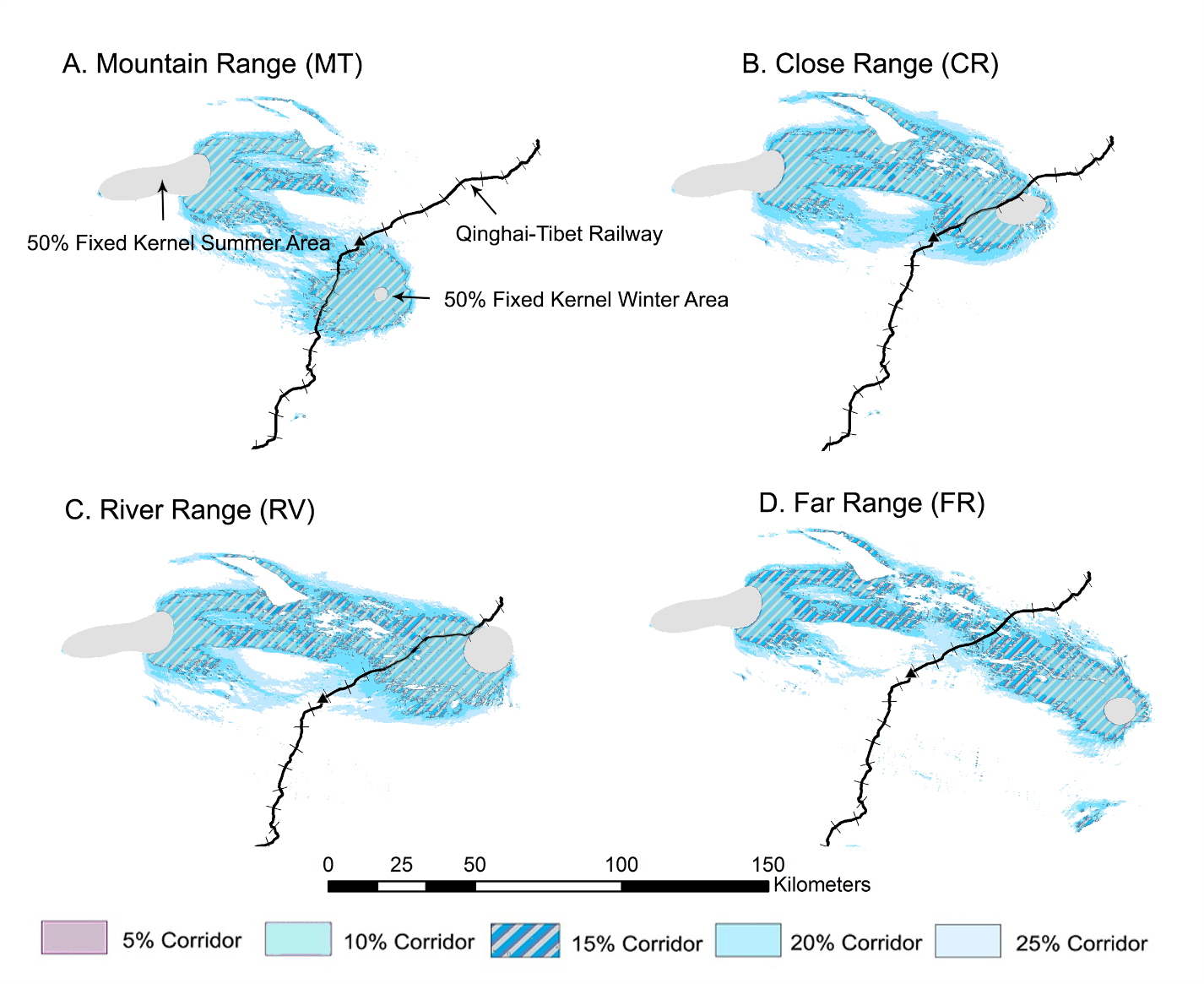


**Fig A1 Corridors based on circuit theory thresholds of 5%, 10%, 15%, 20%, and 25%.**
