# Supplementary material for: Railway underpass location affects migration distance in Tibetan antelope (Pantholops hodgsonii)
Source: PLoS One. 2019 Feb 4;14(2):e0211798. doi: 10.1371/journal.pone.0211798 (PMC6361455; doi:10.1371/journal.pone.0211798)
Supplement: S2 Appendix — (DOCX) [file pone.0211798.s002.docx]

**S2 Appendix: least-cost paths based on resistance surfaces generated based on different resistance surfaces**

To test result sensitivity to resistance settings, we created least cost paths based on resistance surfaces generated only from elevation and slope layers in addition to the one based on both layers (elevation+slope, elevation only, slope only).

We calculated distances from Argos locations to the modelled least cost path and the underpass. Least cost path results are slightly different between resistance surface used (Fig A.2: A – D). However, these differences do not alter our conclusion on distances calculated (Fig A.2: E – F). Compared to the elevation+slope models, slope-only and the elevation-only models showed a stronger negative correlation between the antelopes’ distance to the railway and the modelled corridor. Spearman rank test (r) for the slope only model was -0.52 (p <0.01) and -0.47 (p<0.01) for the elevation only model. Therefore, using both elevation and slope layers for resistance surface is able to generate robust models to estimate theoretically energy-efficient corridors.


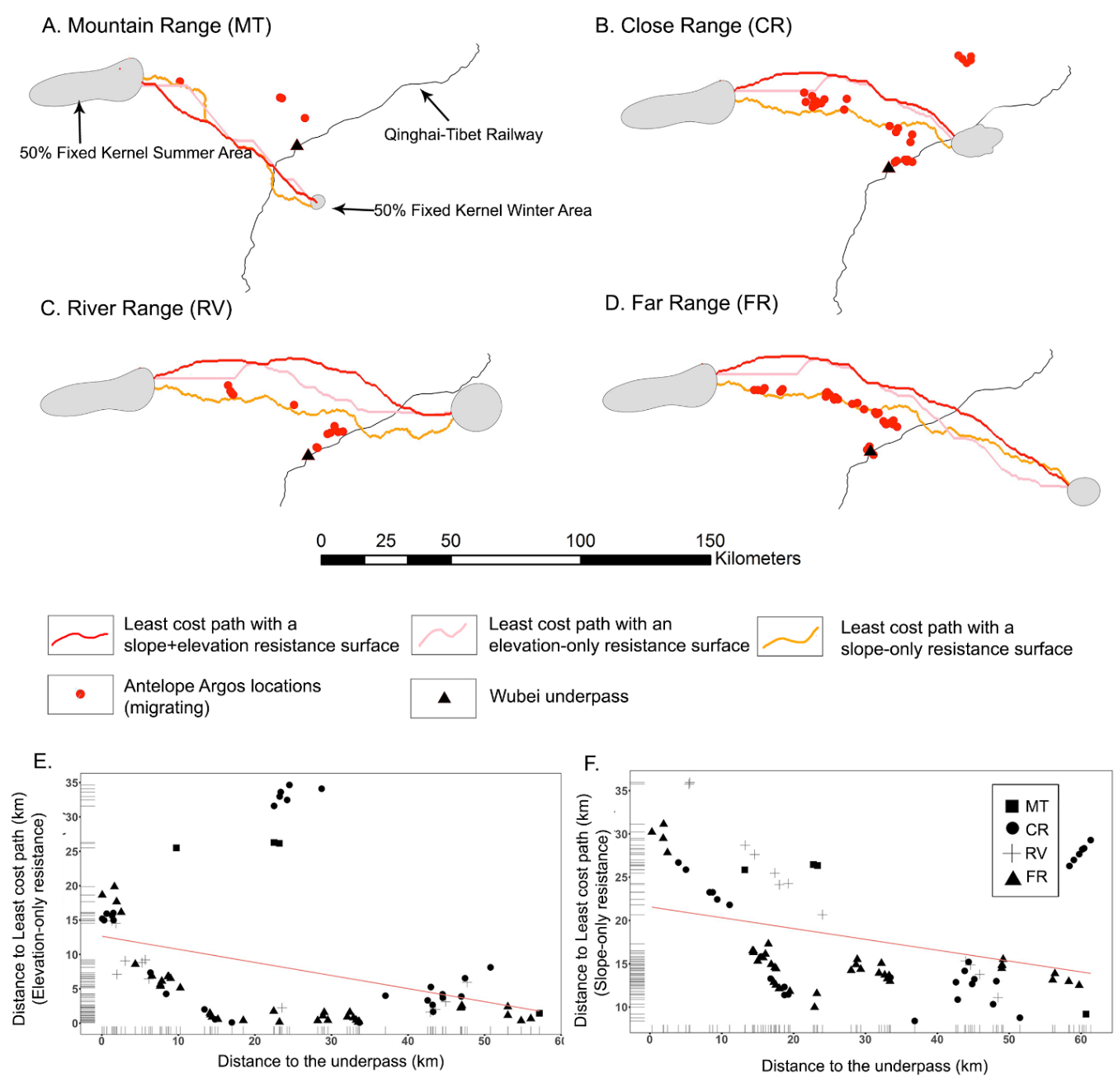


**Fig A2: Least-cost path modeling based on different resistance surfaces.** A-D: Modelled least-cost path based on three resistance surface (slope+elevation, slope-only, and elevation-only); E-F: Distance to the railway vs. distance to least-cost path based on elevation only and slope only resistance surface.
