# Supplementary material for: Railway underpass location affects migration distance in Tibetan antelope (Pantholops hodgsonii)
Source: PLoS One. 2019 Feb 4;14(2):e0211798. doi: 10.1371/journal.pone.0211798 (PMC6361455; doi:10.1371/journal.pone.0211798)
Supplement: S3 Appendix — (DOCX) [file pone.0211798.s003.docx]

**S3 Appendix: Spatial bias and data accuracy in Argos data**

The Argos data interval is irregular, ranging from 0 to 3 points every day. To ensure the analysis quality, we deleted locations with spatial errors >= 1.5 km. For each individual, years with > 2 months of continuous data missing were removed in order to retrieve yearly migration cycles. As shown in main manuscript Fig 3, the remaining locations during the migration season all lie to the west of the railway. This is a result from a combination of Argos data spatial bias, missing data, and deletion of data with low accuracy.

To confirm the spatial error of Argos relocations did not significantly bias the distance calculation, we plot the migration locations with a buffer indicating the spatial error (radius of error) relative to the least-cost paths and the Wubei underpass location. According to Argos data manual (http://www.argos-system.org/manual/3-location/34_location_classes.htm), LC3, LC2, and LC1 have an estimated error (or the radius of error) of < 250m, 250m < < 500m and 500m < < 1500m, respectively. We created approximate (isotropic) circle buffers with the radius at the larger end of the error range (i.e. 250m, 500m and 1500m, respectively). Figure A.3 visually shows that the spatial error should not affect the general patterns of the relative locations of animal migration route and the least-cost path.

We acknowledge that the Argos position error is better represented by an ellipse rather than by a circle due to the satellite’s polar orbit [1]. The newly available Kalman filtering method is able to reduce mean error by 10% to 63%, especially for low quality relocations LC A and B [2]. In our case, we only retained relocations that have at least 4 satellite messages (LC 3, 2, 1) and discarded LC A and B. Our main goal is to showcase relative locations between animal migration route and landscape features (railway, underpass, topographically-determined optimal migration route), rather than to retrieve fine resolution animal locations for movement modeling (e.g. [3]). We thus believe a radius error representation is adequate for our purpose.


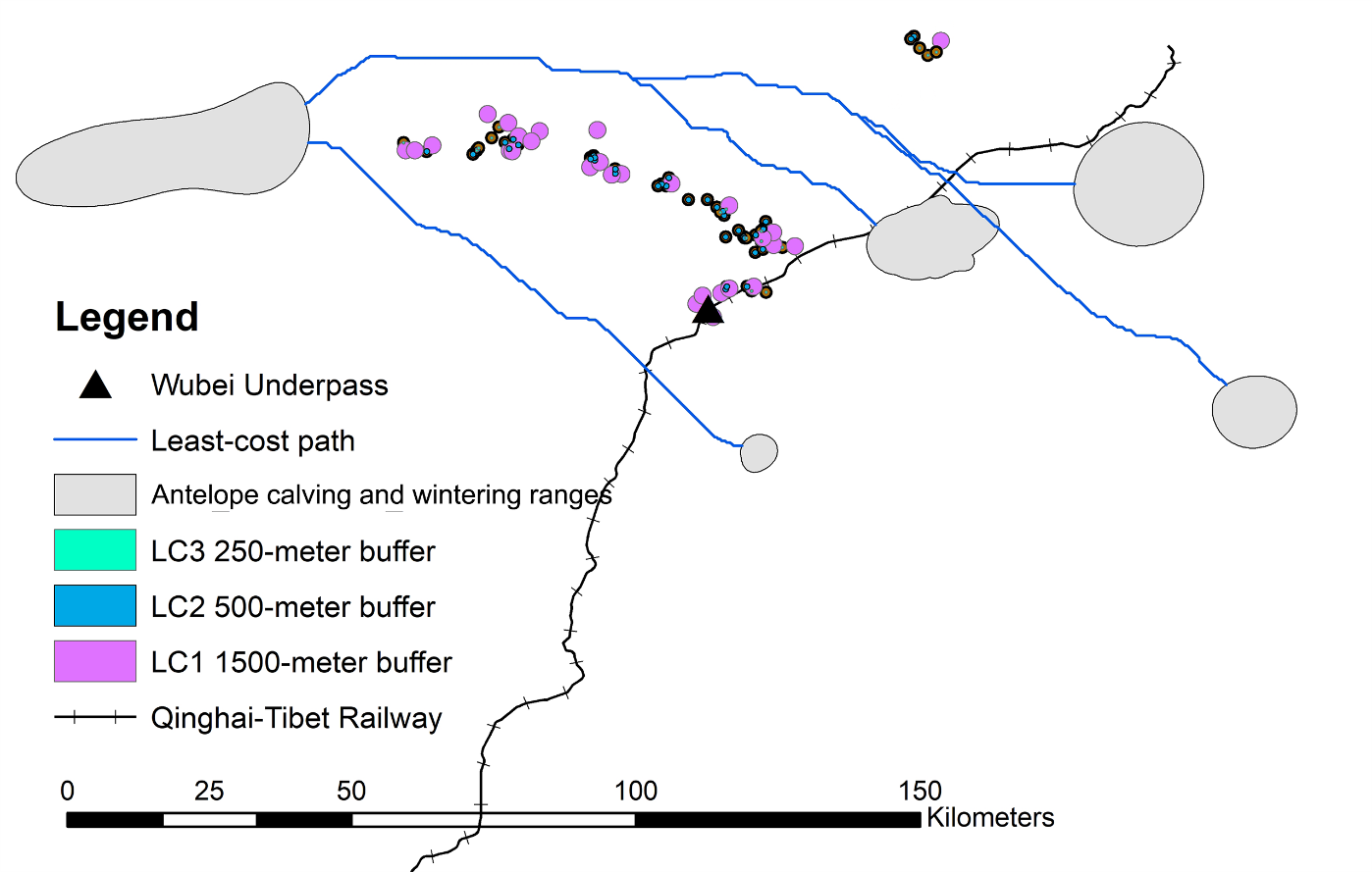


**Figure A3: Migrating Argos location with error buffers.**

1. Satellites CL. Argos user’s manual. Collecte localisation satellites, Ramonville-Saint-Agne, France. 2011 Jun.

2. Lopez R, Malardé J, Royer F, Gaspar P. Multiple-Model Kalman Filtering. 2014;52: 4744–4755.

3. Mcclintock BT, London JM, Cameron MF, Boveng PL. Modelling animal movement using the Argos satellite telemetry location error ellipse. Methods Ecol Evol. 2015;6: 266–277.
