# Supplementary material for: Railway underpass location affects migration distance in Tibetan antelope (Pantholops hodgsonii)
Source: PLoS One. 2019 Feb 4;14(2):e0211798. doi: 10.1371/journal.pone.0211798 (PMC6361455; doi:10.1371/journal.pone.0211798)
Supplement: S1 Table — (DOCX) [file pone.0211798.s004.docx]

**S1 Table: Summary of All Corridor Models Generated in the Study**

| Model #^*^ | Location in the Paper | Modeling Method | Resistance Surface Factors | Purpose |
| --- | --- | --- | --- | --- |
| 1 | Main Manuscript | Circuit Theory | Waterbody + Slope + Elevation | Main Analysis  Main Analysis |
| 2 | Main Manuscript | Least-Cost Path | Waterbody + Slope + Elevation |  |
| 3 | S1 Appendix | Circuit Theory | Waterbody + Slope + Elevation | Find proper current threshold for circuit theory corridor modelling |
| 4 | S1 Appendix | Circuit Theory | Waterbody + Slope + Elevation |  |
| 5 | S1 Appendix | Circuit Theory | Waterbody + Slope + Elevation |  |
| 6 | S1 Appendix | Circuit Theory | Waterbody + Slope + Elevation |  |
| 7 | S1 Appendix | Circuit Theory | Waterbody + Slope + Elevation |  |
| 8 | S2 Appendix | Least-Cost Path | Waterbody + Slope | Test result sensitivity to different resistance surfaces |
| 9 | S2 Appendix | Least-Cost Path | Waterbody + Elevation |  |

^*^ Note that each model contains four corridors generated for each of the four calving/wintering pairs (as four different starting/ending pairs)
