# Supplementary material for: Railway underpass location affects migration distance in Tibetan antelope (Pantholops hodgsonii)
Source: PLoS One. 2019 Feb 4;14(2):e0211798. doi: 10.1371/journal.pone.0211798 (PMC6361455; doi:10.1371/journal.pone.0211798)
Supplement: S2 Table — The model was fit in a nonlinear mixed-effect model framework for each individual in each year. Migration cycles are labeled using individuals’ ID and 2-digit year (“Individual_year”). (DOCX) [file pone.0211798.s005.docx]

**S2 Table: Summary of migration parameters estimated from net-square displacement models.**

The model was fit in a nonlinear mixed-effect model framework for each individual in each year. Migration cycles are labeled using individuals’ ID and 2-digit year (“Individual_year”).

| ID_Year | Wintering site^a^ | Wintering points # | Calving points # | Migration points # | δ (km^2^) ^b^ | θ_s_ (day of year) | θ_r_ (day of year) |
| --- | --- | --- | --- | --- | --- | --- | --- |
| 39_11 | CR | 29 | 33 | 4 | 27104.26 | 161.82 | 201.12 |
| 39_13 | CR | 33 | 7 | 4 | 26234.32 | 160.87 | 199.75 |
| 40_11 | CR | 24 | 17 | 3 | 15767.13 | 147.7 | 186.08 |
| 35_10 | FR | 108 | 31 | 18 | 66554.74 | 169.52 | 201.69 |
| 44_11 | FR | 74 | 37 | 7 | 51128.29 | 174.15 | 203.23 |
| 44_13 | FR | 60 | 31 | 12 | 34231.36 | 153.62 | 200.52 |
| 38_10 | MT | 96 | 22 | 15 | 20663.12 | 159.29 | 195.04 |
| 41_11 | RV | 173 | 38 | 20 | 30084.65 | 170.73 | 194.01 |
| Sum |  | 597 | 216 | 83 |  |  |  |
| ­Average |  |  |  |  | 33970.98 | 162.21 | 197.68 |

^a^FR – Far range; MT – Mountain range; CR – Close range; RV – River range

^b^δ– Squared migration distance; θs – Day of year of the starting trip; θr– Day of year of
 the returning trip

^c^Average starting trip duration (φs) is 8.35 days and returning trip duration (φr) is 5.19 days for the eight migration cycles.
