# Supplementary material for: Railway underpass location affects migration distance in Tibetan antelope (Pantholops hodgsonii)
Source: PLoS One. 2019 Feb 4;14(2):e0211798. doi: 10.1371/journal.pone.0211798 (PMC6361455; doi:10.1371/journal.pone.0211798)
Supplement: S3 Table — Migration distance estimated by net-square displacement (NSD) and by directly connecting Argos relocations with Wubei underpass as one point represents actual migration conducted by antelopes. Distance estimated by least-cost path (LCP) represents optimal migration. Prolonged distance is calculated by subtracting LCP distance from NSD and Argos distance, respectively. (DOCX) [file pone.0211798.s006.docx]

**S3 Table: Migration distance (km) and detour distance for each individual-year migration cycle.**

Migration distance estimated by net-square displacement (NSD) and by directly connecting Argos relocations with Wubei underpass as one point represents actual migration conducted by antelopes. Distance estimated by least-cost path (LCP) represents optimal migration. Prolonged distance is calculated by subtracting LCP distance from NSD and Argos distance, respectively.

| ID_Year | Wintering site | NSD | | Argos | LCP | NSD-LCP | Average by wintering site | Argos-LCP | | Average by wintering site |
| --- | --- | --- | --- | --- | --- | --- | --- | --- | --- | --- |
| 39_11 | CR | 164.63 | 229.17 | | 116.66 | 47.97 | 34.06 | | 112.51 | 110.57 |
| 39_13 | CR | 161.97 | 163.60 | | 116.66 | 45.31 |  |  | 46.94 |  |
| 40_11 | CR | 125.57 | 288.92 | | 116.66 | 8.91 |  |  | 172.26 |  |
| 35_10 | FR | 257.98 | 271.21 | | 190.46 | 67.52 | 32.58 | | 80.75 | 49.80 |
| 44_11 | FR | 226.12 | 252.02 | | 190.46 | 35.66 |  |  | 61.56 |  |
| 44_13 | FR | 185.02 | 197.54 | | 190.46 | -5.44 |  |  | 7.08 |  |
| 38_10 | MT | 143.75 | 180.56 | | 98.37 | 45.38 | 45.38 | | 82.19 | 82.19 |
| 41_11 | RV | 173.45 | 250.98 | | 148.79 | 24.66 | 24.66 | | 102.19 | 102.19 |
| Average |  | 179.81 | 229.25 | | 146.07 | 33.75 | 34.17 | | 83.19 | 86.19 |
